# Safer Healthcare Substitution Needs Life-Cycle Evidence: Lessons from the Post-amalgam Transition

**DOI:** 10.1021/acs.est.6c13088

**Published:** 2026-10-01

**Authors:** Marcin Mikulewicz, Katarzyna Chojnacka

**Affiliations:** † Division of Facial Abnormalities, Department of Dentofacial Orthopaedics and Orthodontics, Wroclaw Medical University, Krakowska 26, 50-425 Wroclaw, Poland; ‡ Department of Advanced Material Technologies, Faculty of Chemistry, Wroclaw University of Science and Technology, Smoluchowskiego 25, 50-372 Wroclaw, Poland

**Keywords:** healthcare substitutions, dental amalgam, safe and sustainable

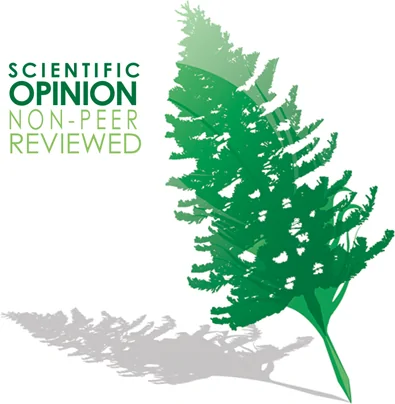

Replacing a hazardous material is not the same as designing a safe and sustainable substitute. A transition can begin with a justified decision to remove a known hazard, yet a phase-out milestone does not establish that the substitute is safe and sustainable. It does not show what replaces the incumbent, how the substitute performs, or where its constituents travel across the life cycle. Major healthcare substitutions should be treated as prospective environmental decisions, not product swaps completed once clinical acceptability is shown.

The global transition away from dental amalgam illustrates the gap. In 2025, decision MC-6/3 under the Minamata Convention set a 2034 phase-out date for the manufacture, import, and export of dental amalgam, excepting use that a dental practitioner considers necessary based on patient needs.^1^ A resin-based composite is a principal substitute for direct restorations. A 2026 systematic review found that both amalgam and non-amalgam restorative materials are associated with environmental impacts, but confidence in comparative conclusions is limited. Study designs and outcome measures are heterogeneous, and comparative data sparse.^2^ A systematic review of polymers in oral healthcare documents polymer waste, monomeric eluates, and micro- and nanoscale particles but concludes that the evidence is limited and extrapolation to environmental effects is uncertain.^3^ This is not a verdict against composite, nor evidence that it rivals mercury; it suggests that substitution has advanced faster than the comparative evidence needed to evaluate it.

The lesson extends beyond dentistry. Mass flow alone is an insufficient basis for prioritization. Even a small flow can warrant attention if its constituents are biologically active, persistent, or mobile, or when releases are repeated, though detection alone does not establish unacceptable risk. Comparison should begin with the clinical function to be delivered, not a one-for-one chemical replacement. Functional substitution can reveal options such as prevention, repair, longer product life, process change, or a different material architecture and keeps candidates from being judged against only the hazard that disqualified the incumbent.^4^ None of this is new. Functional substitution and assessment of alternatives have raised these issues across chemicals policy. What is distinctive in healthcare is that the function is clinical, the exposed population is a patient, and the rules governing the switch were written for safety, not the environment.

Within those rules, a defensible substitution decision asks four questions before adoption at scale. What function is essential, and can demand be reduced without compromising care? How do the incumbent and the candidates compare in clinical performance, intrinsic hazard, plausible exposure, and transformation products? What burdens arise from manufacture through use, maintenance, reuse, recycling, and disposal? What minimum evidence and postmarket surveillance are needed, with what predefined findings triggering redesign or control? A shared clinical functional unit and consistent life-cycle assumptions make the first three tractable. The fourth is most often left open, and most specifiable. A minimum evidence set can be published: declared composition, release data generated under clinically realistic use and removal conditions, service-life evidence in that functional unit, and a documented end-of-life route. Triggers can be fixed in advance: a release above the assumed value, a shorter service life, an unanticipated transformation product, and an unavailable end-of-life route, each paired with the response it activates and a responsible party.

The framework must complement, and not disrupt, the regulatory landscape around health care. The clinical utility hurdle cannot be compromised. A clinically inferior but environmentally better substitute is not a substitute at all. Device regulation and biocompatibility standards (EU Medical Device Regulation 2017/745, U.S. Food and Drug Administration premarket pathways, ISO 10993) already require rigorous characterization of component materials and exposures, but the exposure addressed is clinical, not environmental. A 2026 overview of rules in the EU found that while there are several requirements addressing ecodesign, packaging, and waste, as well as financial incentives to promote sustainability, these lie in other legislative instruments, not explicitly in the MDR, while the latter’s requirements for postmarket surveillance focus on safety-related reporting of incidents.^5^ There is no formal mechanism to feed environmental findings into device regulations. Fortunately, most devices are already required to have technical documentation, clinical evaluation and postmarket clinical follow-up plans, and unique device identifiers. Adding additional composition, release, and end-of-life information would mean further fields on dossiers that manufacturers must compile.

Procurement and clinical practice are where such requirements would bite fastest. Health systems buy through centralized and public tenders, group purchasing organizations, and multiyear framework agreements, so award criteria propagate quickly. Hospitals and dental services are large, contract-based buyers, not dispersed consumers. Tenders can make that evidence set, plus a take-back route, weighted award criteria. The largest barrier is the lowest-price award; others are clinician preference for familiar products, practice fragmentation outside any tender, cost and liability, and a clinical autonomy that no purchasing rule should override. Remedies exist. Longevity and failure data in clinical registries and insurance claims are a ready-made source of service-life evidence. Amalgam separators show that point-source capture can be mandated and verified even in dispersed practice. Professional bodies can place evidence expectations in guidelines, and reimbursement can reward durability and repair over replacement. The same levers apply beyond dentistry, to plasticized PVC in tubing or inhaled anesthetic gases.

Responsibility is shared. Clinicians can favor prevention, repair, and techniques that reduce excess material and waste; developers can design for durability, material transparency, low constituent release, and feasible recovery or disposal. Standardized tests should simulate clinically realistic use and disposal, report uncertainty, and not rank products on idealized tests. The post-amalgam transition should become a model for learning during substitution, not an argument to delay mercury elimination. Baseline material-flow estimates, targeted monitoring, and transparent reporting can indicate which releases merit action - by redesign, capture, procurement change, or none-provided the assessment stays comparative and proportional. Clinical benefit remains the first gate, but it should not be the only one. A substitution decision is more defensible when clinicians, designers, purchasers, and environmental scientists evaluate function, chemistry, clinical performance, material flows, and end of life before widespread adoption.
